# Evolution of Surface Catalytic Sites on Bimetal Silica-Based Fenton-Like Catalysts for Degradation of Dyes with Different Molecular Charges

**DOI:** 10.3390/nano10122419

**Published:** 2020-12-03

**Authors:** Ivalina Trendafilova, Andraž Šuligoj, Alenka Ristić, Nigel Van de Velde, Goran Dražić, Mojca Opresnik, Nataša Zabukovec Logar, Albin Pintar, Nataša Novak Tušar

**Affiliations:** 1 National Institute of Chemistry, Hajdrihova 19, 1000 Ljubljana, Slovenia; Ivalina.Trendafilova@orgchm.bas.bg (I.T.); andraz.suligoj@ki.si (A.Š.); alenka.ristic@ki.si (A.R.); nigel.van.de.velde@ki.si (N.V.d.V.); goran.drazic@ki.si (G.D.); mojca.opresnik@ki.si (M.O.); natasa.zabukovec@ki.si (N.Z.L.); albin.pintar@ki.si (A.P.); 2 Institute of Organic Chemistry with Centre of Phytochemistry, Bulgarian Academy of Sciences, G. Bonchev Blvd. Bld. 9, 1113 Sofia, Bulgaria; 3 Faculty of Chemistry and Chemical Technology, University of Ljubljana, Večna pot 113, 1000 Ljubljana, Slovenia; 4 Graduate School, University of Nova Gorica, Vipavska 17, 5000 Nova Gorica, Slovenia

**Keywords:** Cu-Mn silica-supported catalyst, Cu and Mn oxide nanoclusters, Cu and Mn oxide nanoparticles, nanocomposites, direct synthesis, extraction-calcination, calcination, Fenton-like dyes degradation, molecular charge-depended dyes degradation, water remediation

## Abstract

We present here important new findings on the direct synthesis of bimetal Cu-Mn containing porous silica catalyst and the effects of structure-directing agent removal from the prepared nanomaterial on the evolution of surface catalytic sites. The extraction-calcination procedure of the structure-directing agent removal led to the formation of Cu and Mn oxo-clusters and Cu and Mn oxide nanoparticles smaller than 5 nm, while the solely calcination procedure led to the mentioned species and in addition to the appearance of CuO nanoparticles 20 nm in size. Catalysts were tested in the Fenton-like catalytic degradation of dyes with different molecular charge (cationic, anionic, and zwitterionic) as model organic pollutants in wastewater at neutral pH. Significantly faster degradation of cationic and anionic dyes in the first 60 min was observed with the catalyst containing larger CuO nanoparticles (>20 nm) due to the less hindered generation of ^•^OH radicals and slower obstructing of the active sites on the catalysts surface by intermediates. However, this was not found beneficial for zwitterionic dye with no adsorption on the catalysts surface, where the catalyst with smaller Cu species performed better.

## 1. Introduction

Toxic organic compounds are one of the most important classes of pollutants in wastewater and a major source of environmental contamination. In the last few decades, many academic and industrial research studies have been focused on developing new highly active and stable catalysts for selective oxidation and total degradation of organic substrates [1,2,3]. Advanced oxidation processes (AOPs) generally require metal species (ions, oxides, complexes) as a catalyst in combination with cost-effective, environmentally friendly oxidizing agents such as O_2_ or H_2_O_2_, which decompose organic compounds (dyes, antibiotics, pesticides, etc.) to H_2_O, CO_2_ and non-harmful inorganic species. The most commonly used AOP in the industry is the so-called Fenton process, a homogeneous catalytic process using iron salts as catalysts [4]. However, its application as a homogeneous catalytic process is limited because of its drawbacks such as (i) optimum efficiency is typically achieved under acidic pH values (pH = 3), which requires large amounts of acid to be used, (ii) formation of large amounts of ferrous iron sludge, (iii) the presence of iron ions in the effluents after the reaction, and (iv) the removal of the catalyst from the system is difficult.

To solve these problems the main research focus is aimed at the development of heterogeneous Fenton-like AOP catalysts based on transition metals like Al, Ce, Cr, Co, Mn, and Cu working under neutral pH values [5,6]. Transition metals in the form of single atoms, nanoclusters, or nanoparticles on porous supports—the so-called supported catalysts—are promising materials for heterogeneous catalysis as they could mimic catalyst performance in the homogeneous catalytic process [7]. Moreover, the design of supported catalysts with more than one transition metal is a premise for increasing their activity, selectivity, and application in different catalytic reactions [8]. Stable, supported redox-active metal species are immobilized onto solid supports by various techniques such as impregnation onto inorganic carriers, anchoring a ligand of a transition metal complex onto the surface of an inorganic or organic carrier, entrapment of metal complexes within the cavities of porous materials (“ship-in-a-bottle” approach), ion-exchanged redox-active and (mostly) Lewis acidic metal cations within zeolites (microporous aluminosilicates) and incorporation of the metal species into the framework of an inorganic oxide during the synthesis of the material (direct synthesis) [7]. The advantage of the direct synthesis approach is usually the generation of active catalytic sites, which are strongly incorporated into the oxide support and the ease of preparation as these are usually one-pot syntheses.

Our previous studies showed that Mn incorporated into a porous silica material is active in Fenton-like reactions for removal of model organic compounds at neutral pH values and room temperature [9]. Other promising materials suitable for AOP heterogeneous catalyst are Cu oxides and nanoparticles, because of their high redox potential, environmental friendliness, low cost [10], and their high efficiency of H_2_O_2_ activation in the neutral pH range [5]. Recently, we prepared Cu-Mn porous silica-supported Fenton-like AOP catalysts with interparticle mesoporosity via direct solvothermal synthesis (Mn incorporation into porous silica support) followed by incipient wetness impregnation (Cu loading onto Mn containing porous silica support) [11]. Cu addition significantly reduced Mn leaching from the porous silica support (from 60 to 30%) and functioned also as additional surface adsorption sites. However, no synergistic effects between Cu and Mn were recorded, meaning that Cu and Mn were acting as separate active entities.

However, every synthesis involving a structure-directing agent requires its subsequent removal from the synthesized material. This has an important influence on the structural properties of the catalytic components and the properties of mesoporous materials [12] Calcination is ubiquitously used for this purpose in the synthesis of mesoporous SiO_2_ materials. Upon calcination, contraction is usually observed and is associated with the formation of Si-O-Si bonds. This provides network stability and creates a fully condensed SiO_2_ phase which provides high water stability due to lack of hydrolysis reactions in aqueous media. However, upon calcination, the textural features such as pore volume, surface area, and surface hydroxylation are reduced as aggregation or even sintering takes place. This is a handicap for colloidal nanoparticles as the mass transport to the active sites can be greatly reduced. As a consequence, mild detemplation methods have been developed such as using oxidation agents (e.g., Fenton reagent) to remove the template [13,14], material equilibration, and drying in a low-surface tension solvent [15] and ultrasound-assisted ion-exchange process using primary alcohols as solvents [16]. The latter technique showed mesoporous materials (MCM-41) kept their hexagonal structure and high surface area after the surfactant extraction and was able to reduce the thermal shrinkage produced by the conventional calcination surfactant degradation.

In our previous work Mn catalytically active component was incorporated into the silica support via direct synthesis followed by post-synthesis impregnation of Cu catalytically active component, only the calcination procedure was used for structure directing agent removal and only methylene blue was tested as a cationic model dye.

In the present study, bimetal Cu-Mn porous silica-supported Fenton-like AOP catalysts with interparticle mesoporosity were prepared by incorporation of both catalytically active components, Cu and Mn, into the support via direct synthesis approach. The effect of the structure-directing agent (herein, triethanolamine) removal procedure over the structural properties and the activity of the catalysts were studied. The template was removed either by extraction followed by calcination or by calcination alone. The catalysts were tested for Fenton-like degradation of dyes with different molecular charge (cationic, anionic, and zwitterionic) as model organic pollutants in wastewater. The procedure of structure-directing agent removal had a large influence on the physico-chemical properties of the catalysts, which consequently affected also catalytic performance.

## 2. Material and Methods

### 2.1. Chemicals

Tetraethylammonium hydroxide (TEAOH, 20%), tetraethyl orthosilicate (TEOS, 98%), manganese(II) acetate tetrahydrate, and copper(II) acetate monohydrate were purchased from Acros Organics, Antwerp, Belgium. Triethanolamine (TEA, 99%) was provided from Fluka, Buchs, Switzerland. Three organic dyes, methylene blue (MB, cationic, Merck, Kenilworth, NJ, United States) plasmocorinth B (PC, anionic) and resazurin (RZ, zwitterionic, both from Sigma–Aldrich, St. Louis, MO, United States) were chosen as model organic pollutants. The chemical structure and properties of the dyes are illustrated in Table 1.

### 2.2. Preparation of the Catalysts

Manganese and copper-containing silica catalysts were prepared according to the previously reported synthesis approach for disordered mesoporous silica KIL [9]. During gel formation, manganese acetate tetrahydrate (Mn(CH_3_COO)_2_·4H_2_O) and copper acetate monohydrate (Cu(CH_3_COO)_2_·H_2_O) with theoretical molar ratios of Mn/Si = 0.01 and Cu/Si = 0.01 were mixed with TEOS (25 g) and stirred for 10 min, followed by addition of triethanolamine (8.86 g) and distilled water (16.4 g). After 30 min of stirring at room temperature tetraethylammonium hydroxide (8.66 g) was added. The solution was stirred with a magnetic stirrer until the formation of a homogeneous gel, which was aged overnight at room temperature and then dried in an oven at 50 °C for 24 h. The obtained solid product was transferred in a Teflon-lined stainless-steel autoclave and heated to 150 °C for 48 h in ethanol. The solid product was filtered and washed with ethanol three times.

The template was removed by using two different procedures: (i) extraction with an ion-exchange process using ammonium nitrate in ethanol as solvent [(NH_4_NO_3_)/EtOH] at 80 °C overnight followed by calcination at 450 °C for 6 h (heating ramp of 1 °C/min) in airflow, and (ii) calcination at 450 °C for 6 h (heating ramp of 1 °C/min) in airflow. The material obtained by extraction-calcination was denoted as CuMnKIL-ExC and material prepared by the calcination method was denoted as CuMnKIL-C.

### 2.3. Materials Characterization

The structural characteristics of the obtained catalysts were investigated by X-ray powder diffraction (XRD). The XRD patterns were recorded on a PANanalytical X’Pert PRO high-resolution diffractometer (Malvern Panalytical Ltd., Almelo, the Netherlands) using CuK_α1_ radiation (1.5406 Å) in the 2θ range from 2 to 80° (100 s per step 0.034°) with a fully opened X’Celerator detector.

The specific surface area, total pore volume, and pore size of the obtained materials were determined from nitrogen physisorption isotherms recorded at −196 °C using the Autosorb iQ3 apparatus (Quantachrome Instruments, Boynton Beach, FL, USA). Before the adsorption analysis, the samples were outgassed under vacuum for 2 h at 200 °C in the port of the adsorption analyzer. The BET specific surface area was calculated from adsorption data in the relative pressure range from 0.05 to 0.2 [15]. The total pore volume was estimated based on the amount adsorbed at a relative pressure of 0.97 [16]. The pore size distributions (PSDs) were calculated from nitrogen adsorption data using an algorithm based on the ideas of Barrett, Joyner, and Halenda (BJH) [17]. The mesopore diameters were determined as the maxima on the PSD for given samples [18,19].

The morphology of the surface and the particle size of the synthesized catalysts were examined by Zeiss Supra^TM^ 35VP (Carl Zeiss, Jena, Germany) field-emission scanning electron microscope (SEM) operating at 1 kV and using a 10 µm aperture. Elemental analysis of all samples was performed by energy dispersive X-ray analysis (EDX) with an INCA Energy system (Oxford Instruments, High Wycombe, UK) attached to the microscope.

Surface charge of the samples was measured by Zetasizer nano ZS instrument (Malvern Panalytical Ltd., Malvern, UK) using electrophoretic light scattering technology in the pH range of 1–12 adjusted by the addition of either 0.1 M NaOH or 0.1 M HCl.

The morphology, size, and shape of the catalyst nanoparticles with the presence and dispersion of manganese and copper species and oxide clusters onto the silica support were investigated by high-resolution scanning transmission electron microscopy (HRSTEM). The analysis was performed on Cs probe corrected scanning transmission electron microscope ARM 200 CF (JEOL Ltd., Tokyo, Japan) with the cold-FEG cathode. The microscope was equipped with dual-EELS system Quantum ER from Gatan and Centurio EDXS system with 100 mm^2^ SDD detector (JEOL Ltd., Tokyo, Japan). For HRSTEM studies a drop of sample suspension in ethanol was placed on a lacey-carbon coated nickel grid and dried at room temperature. To minimize the electron beam-induced damage 80 kV accelerating voltage was used for analysis.

The Raman spectra were recorded in the spectral range from 100 to 1400 cm^−1^ using an Alpha 300 confocal microscope (Witec, Ulm, Germany) that employed a green laser with an excitation wavelength of 532 nm, an accumulation time of 50 s, and a resolution of 4 cm^−1^. For each sample, three different locations were analyzed to verify the spectra.

The X-ray photoelectron spectroscopy (XPS or ESCA) analyses were carried out on the PHI-TFA XPS spectrometer (Physical Electronics Inc, Chanhassen, MN, USA). The analyzed area was 0.4 mm in diameter and the analyzed depth was about 3–5 nm. Sample surfaces were excited by X-ray radiation from the Mg source. During data processing, the spectra were aligned by setting the Si 2p peak at 103.0 eV, characteristic for SiO_2_ compound. To reduce possible charging on the surface, the low energy electron gun was used for charge neutralization. The accuracy of binding energies was about ±0.5 eV. The smallest concentration of elements that can be detected with XPS method (sensitivity) is about 0.5 at. %.

### 2.4. Catalytic Tests

The catalytic wet hydrogen peroxide oxidation (CWHPO) reactions were carried out in a five-necked glass reactor thermostated at 25 °C under continuous stirring (300 rpm) and purging with nitrogen. In a typical run, 250 mL aqueous solution of dye (MB, PC, RZ) with a concentration of 25 mg/L was transferred into the reactor followed by adding 100 mg of the catalyst. The reaction mixture was kept under these conditions for 60 min for adsorption-desorption equilibrium to be established. After that, the oxidizing agent (H_2_O_2_) with a concentration of 3.3% was added. At appropriate time intervals (up to 300 min), 1 mL aliquots were withdrawn and immediately centrifuged at 6000 rpm for 2 min for the removal of catalyst particles. The temporal concentration of dyes was determined using a Lambda 45 UV-Vis spectrophotometer (Perkin Elmer Inc, Waltham, MA, USA) measuring absorbance at 664 nm (MB), 550 nm (PC) and 600 nm (RZ). The catalytic activity of the examined materials in all reactions was tested in triplicate to confirm the reproducibility of results.

## 3. Results and Discussion

### 3.1. Materials Characterization

**Figure 1:**
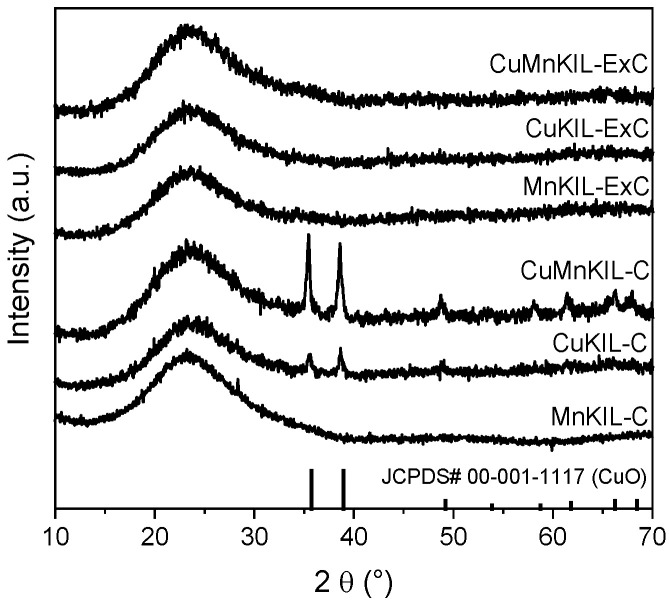
XRD patterns of the obtained materials.

X-ray powder diffraction (XRD) patterns of the investigated materials confirmed an amorphous mesoporous structure of silica seen by the hump in 2θ range of 15–35° (Figure 1). The material prepared by removing the template by calcination (CuMnKIL-C) showed the presence of the monoclinic phase of CuO (20 nm, calculated by the Scherrer equation) as opposed to the sample prepared by extraction-calcination (CuMnKIL-ExC) which showed no presence of CuO phase. For none of the samples, the diffraction peaks characteristic for crystalline manganese oxides or other crystalline copper oxides were observed; however, Cu and Mn oxides were observed in the acquired Raman spectra. This can be explained by the presence of highly dispersed nanosized crystalline Mn and Cu oxides (<5 nm) that are not detectable by the XRD technique.

The elemental compositions of the obtained materials analyzed by energy-dispersive X-ray (EDX) are presented in Table 2. In both samples, the Mn content is approximately the same, while the content of Cu in CuMnKIL-ExC is lower than in CuMnKIL-C, probably due to the washing of Cu species during the extraction procedure. It can be assumed that part of the Cu species is not stabilized onto the silica surface during the catalyst preparation procedure but remains present in the organic part (i.e., the template).

**Figure 2:**
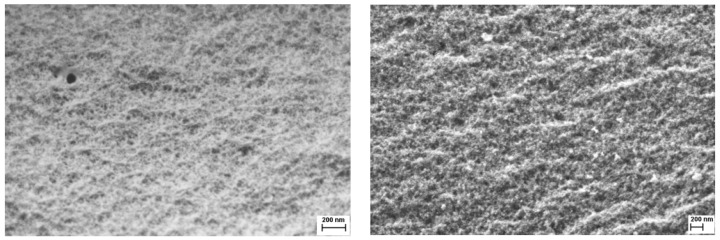
SEM images of CuMnKIL-C (**left**) and CuMnKIL-ExC (**right**) samples.

The SEM images presented in Figure 2 show the morphology of synthesized samples. For both catalysts, formation of the porous structure with interparticle porosity is clearly seen.

**Figure 3:**
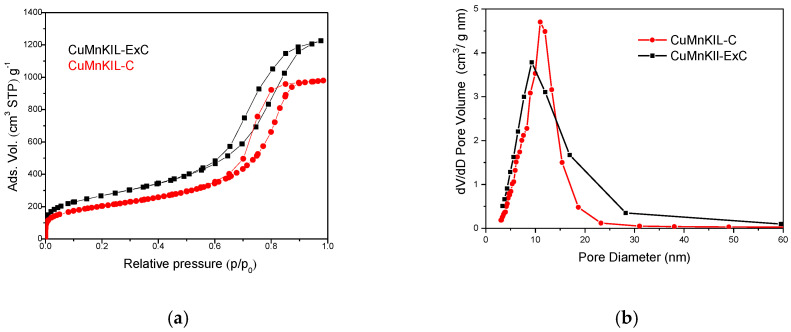
(**a**) Nitrogen sorption isotherms and (**b**) pore size distributions of CuMnKIL samples.

The textural properties of the obtained catalysts were investigated by nitrogen physisorption measurements and are summarized in Table 3. Results show that both materials possess high specific surface area (>700 m^2^/g). CuMnKIL-ExC and CuMnKIL-C samples in Figure 3a show sorption isotherms with a type IV hysteresis loop typical for manganese functionalized KIL silica, showing interparticle mesoporosity [9]. It can be observed that CuMnKIL-C sample possesses less intense hysteresis loop, indicating the presence of larger CuO nanoparticles, which is in accordance with XRD patterns. Less broad pore size distribution (Figure 3b) was determined for the CuMnKIL-C sample. Microporosity, evident in CuMnKIL-ExC, is larger than in CuMnKIL-C due to the utilization of “soft” template removal procedure. Namely, the extraction procedure was chosen for template removal to preserve specific surface area during detemplating [20]. The subsequent calcination step was applied to obtain active species in form of metal oxides. The N_2_-sorption results show that the extraction-calcination procedure indeed leads to higher specific surface area and total pore volume in comparison to the material obtained utilizing calcination alone (Table 3). Ethanol left in the pores after extraction is removed more easily due to its low surface tension (σ ≅ 47.5 and ~24 mN/m for TEA [21] and EtOH [22], respectively), and thus causes less pore volume shrinkage. Additionally, it has been reported that solvent with lower boiling point is released from the pores faster, due to higher fluid drag, caused by the capillary forces acting during relatively quicker drying of EtOH [23].

**Figure 4:**
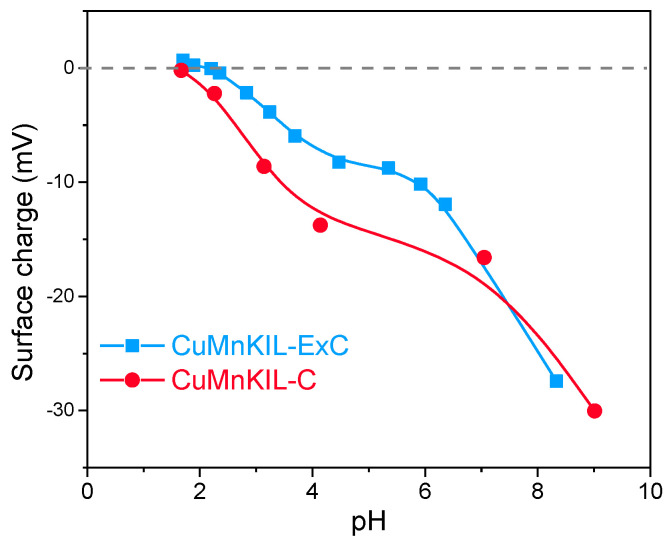
Surface charge data of the catalysts as a function of the pH value of the medium.

The dependence of the catalyst surface charge as a function of pH value is illustrated in Figure 4. At the neutral pH value of the dye-contaminated water (pH = 6–7) all catalysts exhibit negatively charged surface (around −20 mV), which is a premise not only for attracting positively charged pollutants but also for their adsorption because of the strong electrostatic interactions.

In respect of this, three different dyes were chosen as model organic pollutants with positive and negative charge as well as in zwitterionic form at neutral pH value.

**Figure 5:**
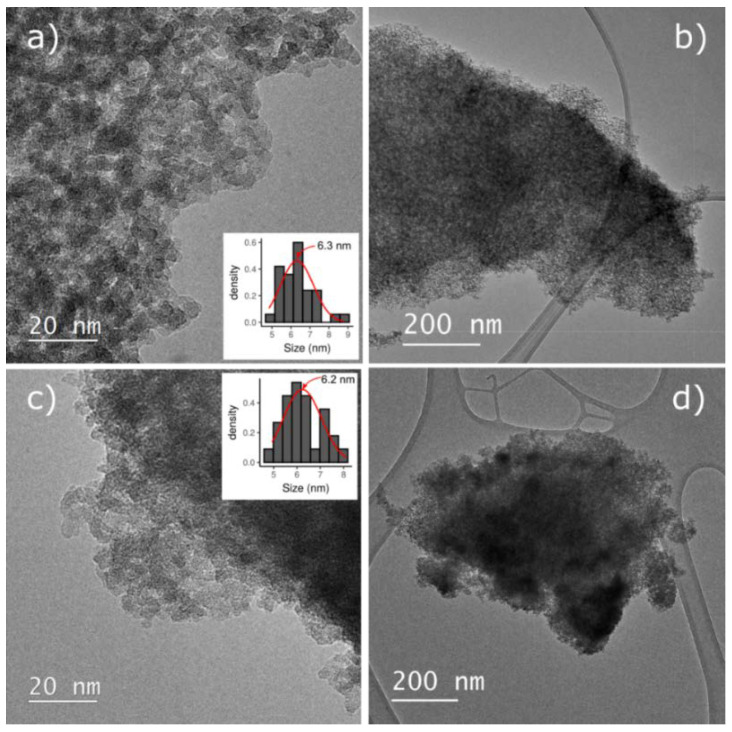
(**a**,**b**) TEM micrographs of CuMnKIL-ExC sample with uniform mesoporous amorphous structure; (**c**,**d**) CuMnKIL-C sample with CuO crystallites (small dark nanoparticles) located on the surface. Inset in (**a**) shows particle size distribution of the amorphous silica particles with a normal curve.

**Figure 6:**
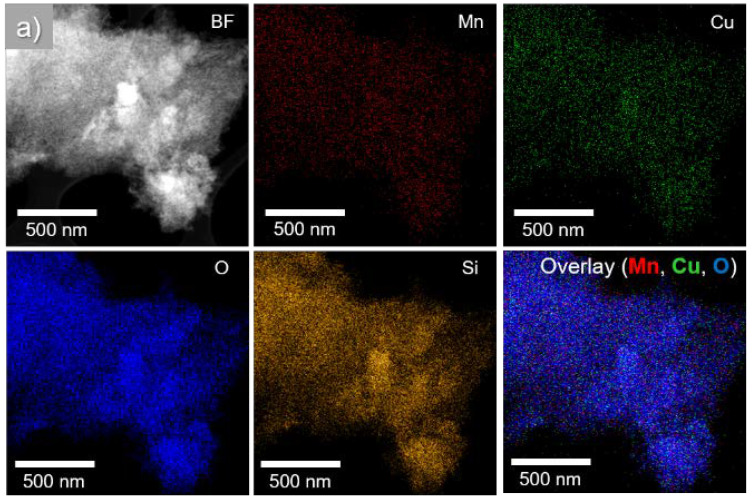

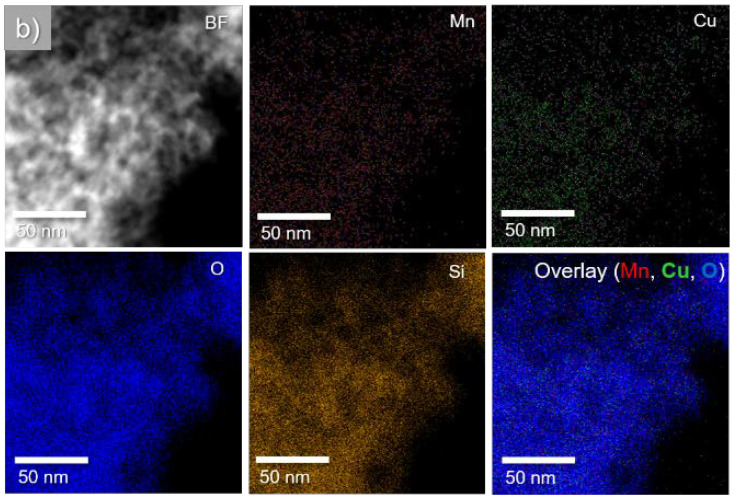
HAADF-STEM images and EDXS mappings of (**a**) CuMnKIL-ExC sample where all elements are uniformly distributed, and (**b**) CuMnKIL-C sample with slight non-uniformity in Cu map.

Transition electron microscopy (TEM) images of CuMnKIL-ExC and CuMnKIL-C samples are presented in Figure 5 and show the formation of amorphous mesoporous structure. While sample CuMnKIL-ExC exhibits uniform structure (Figure 5a,b), in sample CuMnKIL-C (Figure 5c,d) darker areas were detected, probably due to the presence of CuO nanoparticles. EDXS mapping (Figure 6) shows that in the case of the CuMnKIL-ExC sample main elements are randomly distributed, while in the CuMnKIL-C sample the distribution of Cu is slightly inhomogeneous indicating also the presence of CuO nanoparticles 20 nm in size.

However, because TEM images did not unambiguously show the presence of Cu-oxides as they were detected in CuMnKIL-C sample by XRD analysis, Raman spectra were recorded to further investigate the nature of transition metal species in the samples.

**Figure 7:**
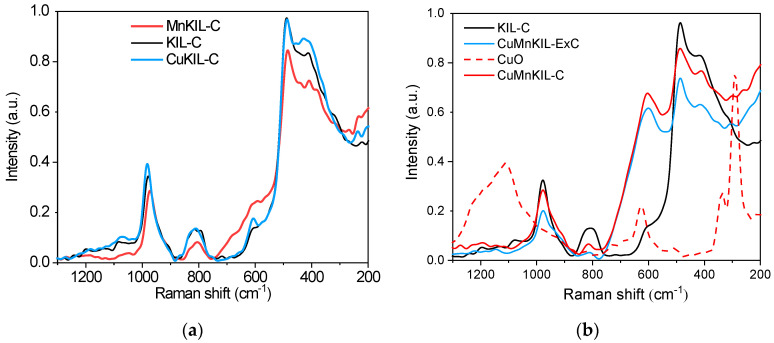
Raman spectra of (**a**) the KIL-C, MnKIL-C, and CuKIL-C samples and (**b**) the CuMnKIL-C, CuMnKIL-ExC, KIL-C, and CuO samples.

In Figure 7a, KIL-C silica (template removal via calcination) exhibits a typical Raman spectrum of the amorphous silica supports like silica gel, SBA-15, KIT-6 [24,25]. Namely, these materials are composed of several common structural motifs including siloxane rings containing from 3 to 6 silicon atoms, Si–O–Si siloxane bridges, and surface silanols with different extents of interaction by hydrogen bonding. These motifs may serve as exchange or grafting sites for transition metal oxides and the structure of the exact exchange site can impact the activity of the resulting transition metal oxide species [24]. The Raman spectrum of KIL-C silica shows the spectral band at 979 cm^−1^, which is associated with the free surface silanols (Si–OH stretching mode), while the spectral band at 810 cm^−1^ is assigned to symmetric Si–O–Si stretching mode. The broad feature at 607 cm^−1^ is attributed to strained 3-member siloxane rings, while bands at 486 and 420 cm^−1^ are assigned to the 4- and 5- or 6-member siloxane rings, respectively.

Raman spectra of calcined MnKIL-C and CuKIL-C samples (template removal via calcination procedure, Figure 7a) indicate the incorporation of manganese and copper in the silica structure, respectively. Manganese and copper exchange at silanol sites and they are as well inserted into 4- and 5- or 6-member siloxane rings. There is an increase in the intensity of a band at around 600 cm^−1^ in both samples, showing on a generation of 3-member rings. Additional bands are observed in both spectra between 230 and 465 cm^−1^ and between 550 and 750 cm^−1^, compared to KIL-C, indicating the presence of Mn and Cu oxides [26,27,28,29]. For example, broad features at 670, 380, and 316 cm^−1^ present in the MnKIL-C sample can be assigned to Mn_3_O_4_ [30,31], while the band at 658 cm^−1^ has been assigned to Mn-oxo clusters [32]. The bands at 410 and 600 cm^−1^ in the spectrum of the CuKIL-C sample show higher intensity than those in the spectrum of the KIL-C, indicating that copper rather inserts into 5-, 6- and 3-member siloxane rings. Additional bands (236, 360, 427, 457, and 657 cm^−1^), due to copper oxides, were found in this spectrum as well.

Figure 7b shows the Raman spectra of CuMnKIL samples with template removal via extraction-calcination (CuMnKIL-ExC) or calcination (CuMnKIL-C). These samples are compared to the calcined KIL-C sample. CuMnKIL-ExC sample shows bands that are dedicated to surface silanols, Si–O–Si siloxane bridges, and 3-, 4-, 5- or 6- member siloxane rings. High increase of the intensity is observed for the 3-member siloxane ring (600 cm^−1^), while the intensities of all other bands decrease, indicating the incorporation of manganese and copper in the KIL-C silica. Besides, several shoulders can be observed (260, 303, 356, 670 cm^−1^), which are not present in the Raman spectrum of the KIL-C sample, showing the presence of nanoparticles of copper and manganese oxides, which are not detected by XRD examination. It can be observed that the band at 600 cm^−1^ in the CuMnKIL-ExC sample is much more intense than in the CuKIL-C or MnKIL-C samples. This band is broad; thus, it is difficult to resolve between bands due to overlapping.

On the other hand, areas rich in larger CuO nanoparticles (spectrum of CuMnKIL-C sample with dashed line denoted as sample CuO—Figure 7b) were found only in the CuMnKIL-C sample. The Raman spectrum of one of these areas shown in Figure 7b (denoted as sample CuO) reveals well-resolved bands at 290, 337, and 627 cm^−1^, typical for CuO [8,27], and an additional broad band at 1110 cm^−1^, which can be assigned to multi-phonon (MP) transition [33], thus indicating that CuO is linked to the silica support via Cu-O-Si bonds [34]. The presence of CuO nanoparticles (20 nm) is in accordance with the acquired XRD patterns. (Figure 1).

**Figure 8:**
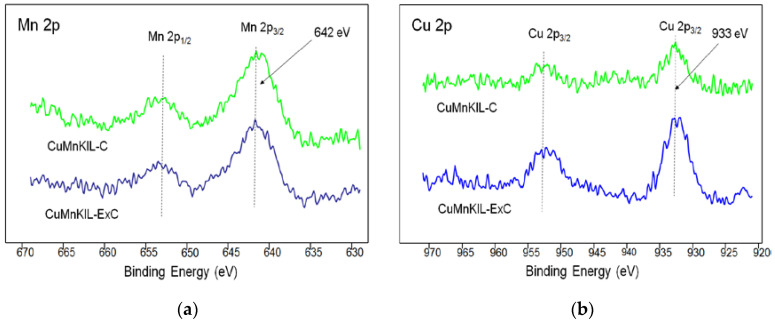
XPS spectra of (**a**) Mn 2p and (**b**) Cu 2p for CuMnKIL samples.

The XPS spectra (Figure 8a) for the Mn 2p of the CuMnKIL samples exhibit two peaks at 642 and 653 eV, corresponding to the Mn 2p_3/2_ and Mn 2p_1/2_ states of Mn_3_O_4_, respectively [35]. XPS technique allows us to distinguish the Cu^2+^-isolated species from Cu^+^ and Cu^0^. The Cu^2+^ has mainly d^9^ character, while Cu^+^ and Cu^0^ species have filled d levels. It is necessary to note that the Cu^+^ ion is difficult to distinguish from Cu^0^ by XPS, but Cu^2+^ species can be easily identified [36]. XPS spectra in Figure 8b reveal two main peaks centered at 933 eV and 952 eV assigned to Cu_2_O. A weak satellite band (944 eV) can be observed, which is characteristic feature for CuO.

**Figure 9:**
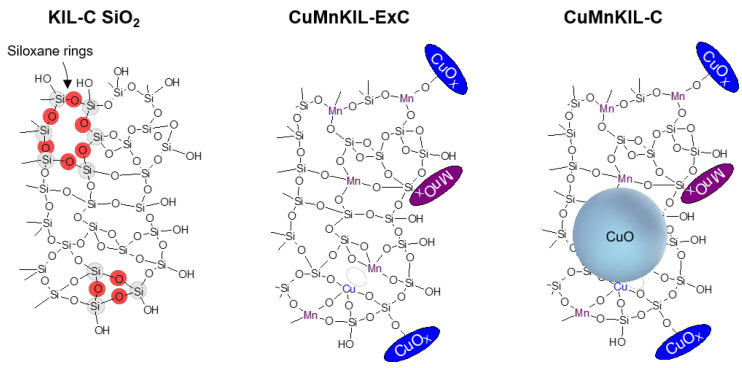
Schematic representation (not to scale) of coordination of metals in the samples of KIL amorphous silica. The 3 and 5 siloxane rings are highlighted in the KIL-C sample. Larger CuO nanoparticles are shown as a sphere on top of the silica lattice in the CuMnKIL-C.

Raman spectra of the CuMnKIL-ExC and CuMnKIL-C samples reveal the presence of manganese and copper incorporated into the KIL silica (amorphous Cu and Mn oxo-clusters), as well as the presence of manganese and copper oxides (crystalline Cu and Mn oxide nanoparticles) which were confirmed also with XPS spectra. It can be therefore concluded that both procedures for template removal (extraction-calcination and calcination) influence the type of species present in both catalysts in two ways (Figure 9): (i) by incorporation of manganese and copper into the silica framework (Cu and Mn amorphous oxo-clusters), and (ii) by the presence of manganese and copper oxides (Cu and Mn crystalline nanoparticles). However, the template removal by extraction-calcination leads to the formation of above mentioned species smaller than 5 nm (XRD invisible), while the template removal by calcination leads additionally to the formation of CuO nanoparticles 20 nm in size (XRD visible).

### 3.2. Catalytic Tests

The catalytic activity of the obtained materials was evaluated in the Fenton-like reactions for removal of model dyes (methylene blue—MB, plasmocorinth B—PC, and resazurin—RZ) with different charge (cationic, anionic, zwitterionic) at neutral pH.

**Figure 10:**
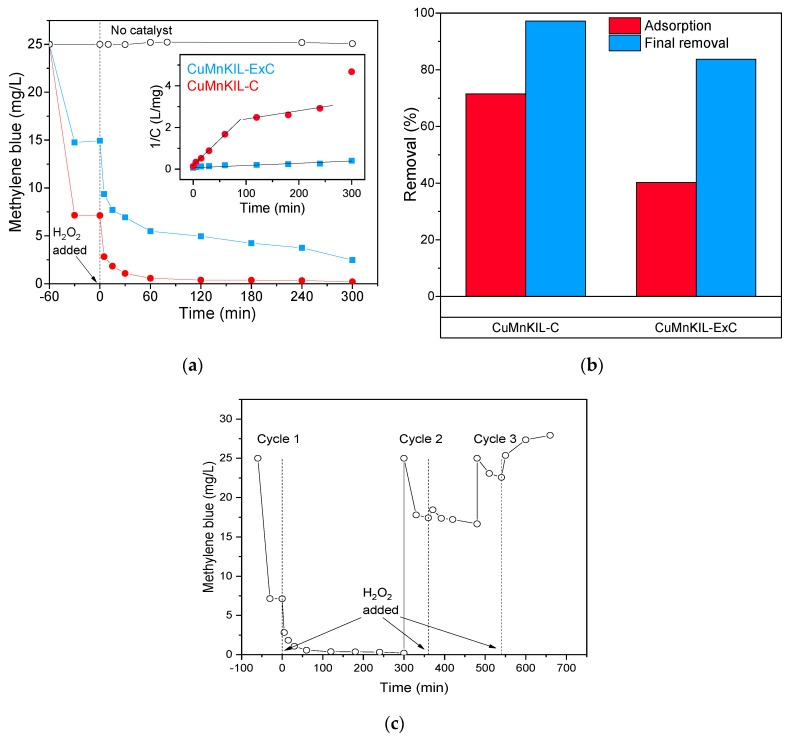
Fenton-like degradation of methylene blue. (**a**) Concentration as a function of time in Fenton-like catalytic reaction, where minute “0” is the time of H_2_O_2_ injection, with the inset showing test for the second-order reaction, *R*^2^ = 0.997 for *t* < 60 in CuMnKIL-C; (**b**) representation of the dark adsorption and the final removal of the dye; (**c**) recycling of the catalyst CuMnKIL-C. All the catalytic tests were repeated at least 3 times to confirm the reproducibility of the obtained results. The error of the analysis was never greater than 5%.

The results from catalytic tests for MB removal (Figure 10a,b) show that the positively charged molecules have a strong affinity towards the negatively charged surface of the catalysts; correspondingly, during the adsorption period, the removal of the dye from the solution is significant. The effect of adsorption is higher for the material prepared by calcination (up to 70%) in comparison to the catalyst prepared by the extraction-calcination procedure (40%) due to the stronger negative charge of the surface of the catalyst (see Figure 4). After the reaction time of 300 min, the concentration of MB in the reaction mixture was decreased by 97% for CuMnKIL-C and 85% for CuMnKIL-ExC. The concentration-time curves of MB depicted in Figure 10a obtained in the presence of both catalysts show initial fast removal for the first 60 min of the reaction and slow removal thereafter. The observed phenomena could be explained by blocking of the active sites of the catalysts, because of adsorption. The differentiation between adsorption (60 min) and final removal (adsorption + 300 min reaction) of the dye is illustrated in Figure 10b. From the presented data it can be seen that the major part of final removal is due to the initial adsorption of the dye on the catalyst surface, which is in accordance with our previously reported results [11]. However, degradation is clearly taking place and it is generally acknowledged [37] that MB degradation via Fenton-like reaction follows three different pathways forming Cl^−^, NO_3_^−^, SO_4_^2−^, and lower molecular weight intermediates such as phenol and benzothiazole. For long term use, catalysts were collected, washed with ~100 mL water, followed by washing with ~20 mL 0.1 HCl, and finally washed with additional ~50 mL water. Recycling test showed the calcined catalyst was not able to keep the activity through successive tests (Figure 10c). Adsorption was successively decreased through recycling as well as the activity after H_2_O_2_ addition. It can be seen that with the third cycle, the catalyst adsorbed considerable amount of dye which was then desorbed in the Fenton-like period without degrading it.

**Figure 11:**
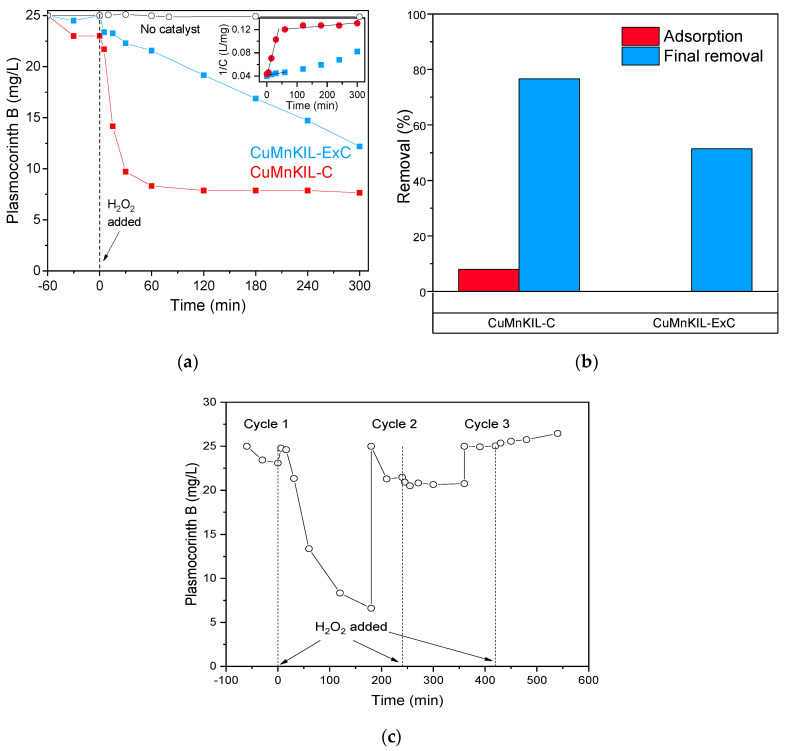
Fenton-like degradation of plasmocorinth B. (**a**) Concentration as a function of time in Fenton-like catalytic reaction with the inset showing the test for the second-order reaction, *R*^2^ = 0.986 for *t* < 60. in CuMnKIL-C; (**b**) representation of the dark adsorption and the final removal of the dye; (**c**) recycling of the catalyst CuMnKIL-C. All the catalytic tests were repeated at least 3 times to confirm the reproducibility of the obtained results. The error of the analysis was never greater than 5%.

Adsorption of negatively charged molecules of anionic PC dye onto the surface of the catalysts shows negligible interaction of the dye with CuMnKIL-C catalyst (8%), while for CuMnKIL-ExC it was not observed at all (Figure 11b). In the case of the Fenton-like reaction for removal of PC the catalysts show a different manner of decomposition of the dye. For the CuMnKIL-C sample, we obtained fast initial decomposition of PC dye up to 65% for the first 60 min followed by a plateau (Figure 11a), which indicates the end of the reaction. For CuMnKIL-ExC we obtained steady decomposition of PC dye up to 50% in 300 min (Figure 10a). The recycling resulted in a decrease in the performance with successive use. The release of adsorbed dye can be seen in the third cycle upon addition of H_2_O_2_ (Figure 11c).

**Figure 12:**
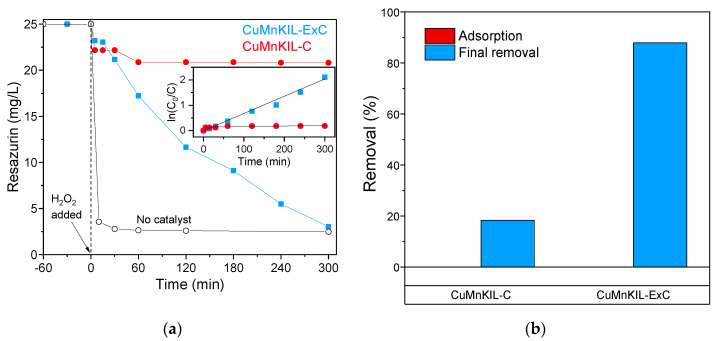
Removal of Resazurin in Fenton-like catalytic reaction. (**a**) Temporal changes in RZ concentration with the inset showing test for the first-order reaction, *R*^2^ = 0.992 in the whole range in CuMnKIL-ExC; (**b**) representation of the dark adsorption and the final removal of the dye. All the catalytic tests were repeated at least 3 times to confirm the reproducibility of the obtained results. The error of the analysis was never greater than 5%.

The results for the removal of zwitterionic dye RZ (Figure 12) show very different behavior compared to cationic MB and anionic PC dyes. CuMnKIL-C shows a fast initial removal of 10% of the RZ dye in the first 60 min and then the reaction ceases. On the contrary, the catalyst CuMnKIL-ExC prepared by extraction-calcination procedure shows a steady decrease in the concentration of the dye throughout the whole 300 min of reaction, and the final degradation of 90% is reached. For both catalysts, no adsorption of RZ on the catalyst surface was observed. The test with no catalyst showed a very fast reaction with the efficiency of 90% already achieved after 30 min. To the best of authors knowledge this is the first Fenton-like degradation of resazurin dye, hence more research is needed to fully understand the mechanism of degradation pathway of this dye.

We have previously found [11] that no synergistic effects between Cu and Mn occurred, meaning that Cu and Mn sites were acting as separate active sites and the main function of Cu addition was to provide additional surface adsorption sites.

Disproportionation of hydrogen peroxide can be considered in terms of the following reduction half-reactions:

H_2_O_2_ + H^+^ + e^−^ → H_2_O → *E*° = +1.68 V (1)

H^+^ + O_2_ + e^−^ → H_2_O_2_ → *E°* = +0.70 V (2)

Therefore, any metal that is to catalyze this reaction has to have suitable binding sites and a standard potential in the range 0.70–1.68 V. The redox reactions of a Mn^3+^/Mn^2+^ couple (*E°* = +1.51 V) in Mn-species in the synthesized samples can thus induce the decomposition of H_2_O_2_ to produce radical species as shown in the following equations [11,38,39]:

≡Mn^2+^ + H_2_O_2_ → ≡Mn^3+^ + ^•^OH + OH^−^ (3)

≡Mn^3+^ + H_2_O_2_ → ≡Mn^2+^ + HOO^•^ + H^+^ (4)

HOO^•^ → H^+^ + O_2_^•−^ p*ka* = 4.8 (5)

OH^−^ + HOO^•^ ↔ O_2_^•−^ + H_2_O^•^ (6)

Dye + ^•^OH, O_2_^•−^ → degradation products → H_2_O + CO_2_ + inorganic acids

On the other hand, the kinetics of the Fenton-like reaction in a perfectly mixed batch reactor can be described as:

(7) $$-r=-\frac{dC}{dt}=k\;C^{n}$$

where *C* stands for the concentration of the dye and *n* represents the reaction order (0–2). The integrated forms of the zero-, first- and second-order rate equations are *C_t_* = *C*_0_ − *k*_0_·*t*, *C_t_* = *C*_0_ exp(−*k*_1_·*t*), and 1/*C* = 1/*C*_0_ + k_2_·*t*, respectively. Fitting of all three reaction rate equations to the collected experimental data showed that the second-order kinetics (*R*^2^ > 0.997 for MB, *R*^2^ >0.986 for PC) best describes the concentration-time profiles with two distinct phases, i.e., a fast degradation in the first 40–60 min, followed by much lower reaction rates. The exception is zwitterionic dye Resazurin, where pseudo-first-order kinetics (*R*^2^ = 0.992) was applied (Figure 11a). During the Fenton-like reaction, dye decomposition is caused by both hydroxyl and hydroperoxyl radicals; however, the latter have significantly lower oxidation power. The above-mentioned shift in global kinetics in dyes removal with Fenton processes has been observed also by others [40,41,42]. In the first stage, the ^•^OH generation by Mn^2+^ is fast due to the abundance of metal centers and H_2_O_2_, while in the second stage it is slower due to the accumulation of Mn^3+^ and low recovery of Mn^2+^ by H_2_O_2_. Similarly, the Cu sites act catalytically by decomposing H_2_O_2_ into ^•^OH via Cu^+^ sites. The data clearly show second-order behavior when adsorption of the dye was present, while zero- or first-order kinetics was observable in cases of no adsorption, thus showing the importance of Mn^2+^/Cu^+^ sites on the surface of the catalysts. Recently, Xu et al. [43] and Santana et al. [42] have also highlighted that the most suitable kinetic model can change from one reaction system to another. Therefore, the pseudo-second-order reaction rate taking into account hydroxyl radicals can be written as:

(8) $$-r=kC_{•OH}^{m}\;C_{dye}^{2}\approx k^{\prime }\;C_{dye}^{2}$$

As mentioned, the ^•^OH concentration is dependent on their generation over Mn^2+^ sites. However, we observed also faster degradation of both cationic and anionic dyes in the first 60 min with CuMnKIL-C catalyst (containing smaller than 5 nm Cu-oxo clusters and Cu oxides together with large CuO nanoparticles) if compared to CuMnKIL-ExC catalyst (containing only smaller than 5 nm Cu-oxo clusters and Cu oxides). We propose this to be a consequence of ^•^OH generation from homolytic H_2_O_2_ cleavage on larger CuO nanoparticles (20 nm in size), which is less influenced by the adsorption of cationic and anionic dyes on the catalyst surface. This in turn results in slower blocking of the active sites on the catalyst’s surface. The high extent of adsorption of the cationic dye compared to the lower level of adsorption of anionic dye is also observed due to the molecular charges of dyes. The opposite behavior was observed in the degradation of zwitterionic dye. We observed much slower degradation of zwitterionic dye in the first 60 min with CuMnKIL-C catalyst in comparison to the degradation of anionic dye using the same catalyst. We assume that the rate of CuMnKIL-C catalyst active sites blocking is much faster in comparison with the anionic dye, which occurs due to the shorter distance between the catalyst surface and the dye/partially oxidized reaction intermediates in the diffuse layer surrounding catalyst particles, because of the lower electrostatic repel between the zwitterionic dye and the catalyst (this might also be a reason for the observed overall first-order kinetics in the case of zwitterionic dye removal). Following this assumption, we can conclude that for the degradation of zwitterionic dye the CuMnKIL-ExC catalyst with smaller Cu oxide species (less than 5 nm in size) is more suitable (Figure 12a) because of the larger surface of CuO ensembles compared to the CuMnKIL-C catalyst containing CuO nanoparticles of 20 nm in size. Thus, the catalysts developed here show promising catalytic features that are comparable to already published materials (Table 4).

To further evaluate the stability of the investigated catalysts, concentrations of leached Mn, and Cu species into the reaction medium after 300 min of reaction were monitored. For both catalysts, Mn leaching was approximately the same, i.e., 4.9 (55.7% of Mn content) and 4.6 (47.9% of Mn content) mg/L for CuMnKIL-ExC and CuMnKIL-C samples, respectively. However, copper addition significantly reduced Mn leaching from the porous silica support if compared to the catalyst that contains only Mn, as it was revealed in our previous study [11]. Still, high amounts of leached Mn can be a problem for the reusability of the catalyst, so that further improvement of the catalyst is necessary to address the leaching problem of Mn. According to the World Health Organization, the legal limit of Mn concentration in drinking water is 0.4 mg/L [48,49]. Subsequent removal of Mn(II) should thus be implemented for practical use; most commonly in wastewater treatment plants this is done by chemical precipitation (e.g., by adding limestone, CaCO_3_) or with biological oxidation mechanisms by using biological aerated filters [50]. In the case of Cu, the leached amount meets the legal restrictions (<2 mg/L) [29] for sample CuMnKIL-C where the observed amount of Cu species after the reactions is 0.4 mg/L (4.5% of Cu content) and is close to the legal limits for sample CuMnKIL-ExC, i.e., 2.3 mg/L (19.8% of Cu content). The impact of leaching of metals can be seen in the recycling tests where the reuse value is poor. The leaching can also explain the fast degradation of the quality of the catalysts upon consecutive reusage. These results show that the direct synthesis approach for active sites immobilization significantly reduces leaching of Cu species in comparison with incipient wetness impregnation method (3.1 mg/L) [11].

## 4. Conclusions

Bimetal Cu-Mn porous silica-supported catalysts with interparticle mesoporosity were prepared by direct incorporation of both catalytic active components, Cu and Mn, into the support via the direct, one-pot synthesis approach. The effect of two structure-directing agent removal approaches on the structural and activity properties of the materials were studied. The results from physicochemical characterization showed that the template removal by extraction followed by calcination (extraction-calcination) led to the formation of the catalyst with high specific surface area and pore volume, where the Cu and Mn oxo-clusters and Cu and Mn oxide nanoparticles with small size (<5 nm) were homogeneously dispersed on the surface of the mesoporous silica support. On the other hand, the removal of the template by calcination alone additionally to the Cu and Mn oxides with small size (<5 nm) led to the sintering of the CuO phase and the formation of 20 nm large particles. Faster degradation of cationic and anionic dyes in the first 60 min with the catalyst containing larger CuO nanoparticles (20 nm in size) if compared with the catalyst containing smaller Cu oxo-clusters and Cu oxides (smaller than 5 nm) is observed. We propose that this is a consequence of the less hindered generation of hydroxyl radicals on larger CuO nanoparticles and thus slower obstructing of the active sites on the catalysts surface. However, after 60 min of the reaction, reduced reaction rates were observed, which is due to even slower ^•^OH production and also slower Mn^2+^ regeneration. For the degradation of zwitterionic dye which causes lower electrostatic repel and attraction (no adsorption) between the dye and catalyst surface, the catalyst with smaller Cu-oxo clusters and Cu oxides (<5 nm in size) is more suitable if compared to the material with larger CuO nanoparticles (20 nm in size). Hence, the control of the size and nature of Cu and Mn species on the surface of mesoporous silica can be achieved by changing the template removal process; this offers new insights into the synthesis of materials for pollutant removal with Fenton-like processes.

Furthermore, the results presented here open up a new avenue for considerations on how to design the surface modification of catalysts to adapt their activity to organic pollutants with different molecular charges.

## Figures and Tables

**Table 1:** Chemical structure and properties of the organic pollutants studied in this work (all dyes 1.5 nm in size).

Dye	Molar Mass (g/mol)	λ_max_ (nm)	Type	Molecular Structure
Methylene blue	319.85	664	cationic	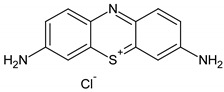
Plasmocorinth B	518.81	550	anionic	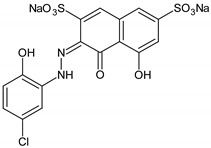
Resazurin	229.19	600	zwitterionic	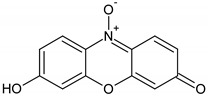

**Table 2:** Elemental composition of the obtained samples from EDX analysis.

Sample	Mn (at. %)	Cu (at. %)	Mn (wt. %)	Cu (wt. %)	Mn/Si	Cu/Si
CuMnKIL-ExC	0.8	0.9	2.2	2.9	0.03	0.03
CuMnKIL-C	0.9	1.4	2.4	4.4	0.03	0.05

**Table 3:** Textural properties of the obtained catalysts.

Sample	BET Surface Area (m^2^/g)	Total Pore Volume (cm^3^/g)	Pore Diameter (nm)
CuMnKIL-ExC	967 ± 1	1.896	9.3
CuMnKIL-C	734 ± 1	1.513	11.1

**Table 4:** Comparison of catalytic results of the catalysts of similar design with the published literature.

Catalyst	H_2_O_2_ (mM)	Pollutant	Pollutant (mg/L)	Efficiency	Reaction Time (min)	Reference
MnO_X_ on Al_2_O_3_	388	MB ^a^	20	99%	400	[44]
Ordered mesoporous MnO_X_	19.6	RhB ^b^	100	90%	150	[45]
γ-MnO_2_	1450	MB ^a^	51	99%	20	[46]
Mn on SiO_2_	97	MB ^a^	25	97%	300	This study
Mn on SiO_2_	97	RZ ^c^	25	90%	300	This study
Mn on SiO_2_	97	PC ^d^	25	65%	300	This study
Mn-Ti-HMS	10	MB & RhB	16 & 24	95%	120	[47]

Dyes’ acronyms: ^a^ Methylene blue, ^b^ Rhodamine B, ^c^ Resazurin, ^d^ Plasmocorinth B.
